# Functional outcomes and toxicity following focal low‐dose‐rate brachytherapy for low–intermediate risk prostate cancer

**DOI:** 10.1002/bco2.70119

**Published:** 2026-02-11

**Authors:** Mohammadmehdi Adhami, Jeremy Cheng, Elliot Anderson, Lloyd Smyth, Cate Davey, Thang Nguyen, Richard O'Sullivan, Andrew Ryan, Nathan Lawrentschuk, Jeremy Grummet, Andrew See

**Affiliations:** ^1^ Department of Urology The Alfred Hospital Melbourne Australia; ^2^ School of Translational Medicine Monash University Melbourne Australia; ^3^ Icon Cancer Centre Melbourne Australia; ^4^ Tissupath Melbourne Australia; ^5^ Department of Urology The Royal Melbourne Hospital Melbourne Australia

**Keywords:** focal brachytherapy, focal therapy, functional outcomes, IIEF‐5, EPIC bowel score, IPSS, prostate cancer

## Abstract

**Objectives:**

We aim to prospectively evaluate functional outcomes and toxicity following focal low‐dose‐rate (LDR) brachytherapy for low–intermediate risk prostate cancer (PCa).

**Patients and Methods:**

LIBERATE is a clinical registry of men treated with focal LDR brachytherapy for low–intermediate risk PCa since September 2019. Follow‐up occurred at 6 weeks and every 3 months thereafter. Outcomes were assessed using validated patient‐reported outcome measures (PROMs): IPSS, Expanded PCa Index Composite (EPIC) Bowel Assessment, and International Index of Erectile Function (IIEF‐5). Adverse events (AEs) were clinically graded per Common Terminology Criteria for Adverse Events v5.0. Minimal important differences (MIDs) were defined as ±3.1 for IPSS, ±5 for EPIC bowel domain, and ±4 for IIEF‐5.

**Results:**

Of 120 patients enrolled, 88/120 (73.3%) had ≥12 months follow‐up, and 79/88 (89.7%) completed PROMs with a median (IQR) follow‐up of 33 (26–41) months. At 6 weeks, a transient, statistically and clinically significant increase in IPSS was observed, which returned to baseline by 12 months (median IPSS: 6 at baseline, 12 at 6 weeks, 6 at 12 months). EPIC bowel scores showed no significant changes, with 72 (91.1%) patients having rectal spacer. Among 66 sexually active men, 40 (60.6%) had no or mild erectile dysfunction (ED) at baseline, with a median IIEF‐5 score of 22.5, decreasing to 22 at 6 weeks and plateauing at 21 at 6 months, none meeting MID criteria. For the 26 patients with mild to severe ED at baseline, the median IIEF‐5 score declined from 10 at baseline to 5 at 6 months, with partial recovery followed by a drop to 5.5 at 2.5 and 3 years. Declines at 6 months and beyond 2 years were both statistically and clinically significant. No Grade ≥3 AEs were reported.

**Conclusion:**

Focal LDR brachytherapy is associated with favorable functional outcomes and minimal toxicity. Further studies are required to evaluate long‐term results.

## 1 INTRODUCTION

Prostate cancer is the second most frequently diagnosed cancer among men worldwide.^1^ Worldwide, approximately 70% of men are diagnosed with localised prostate cancer amenable to curative‐intent whole‐gland treatments, whereas in Australia and New Zealand this proportion is closer to 90%, with the majority presenting with disease confined to the prostate.^2^, ^3^ However, whole‐gland therapies carry a considerable risk of genitourinary (GU), sexual and gastrointestinal (GI) side effects.^4^ Efforts for earlier diagnosis through opportunistic or structured screening have resulted in an increased rate of lower‐risk, lower‐volume diseases that would have otherwise been identified at more advanced stages.^5^ Active surveillance is a suitable approach for many of these patients; however, it has limitations. This has provided the opportunity to consider the novel paradigm of focal therapy, as treating these patients with conventional whole‐gland therapies may result in significant overtreatment and long‐term quality‐of‐life implications without any clear survival advantage.^6^ Focal therapy provides a midway alternative between active surveillance and whole‐gland radical treatments. It involves treating only the index lesion and ongoing active surveillance of the unaffected gland, which reduces the risk of harm to surrounding anatomical structures and thereby minimises toxicity.

While various energy sources are under investigation, the optimal modality for achieving superior functional and oncological outcomes remains unclear.^7^ Low‐dose‐rate (LDR) brachytherapy is a well‐established treatment in the whole‐gland setting for low‐intermediate risk prostate cancer.^8^ However, evidence on patient outcomes following focal LDR brachytherapy is particularly lacking, with only 10 studies published to date, the largest of which included only 50 patients.^5^

Given the limited long‐term data, the 2024 European Association of Urology (EAU) guidelines recommend offering focal therapy exclusively within the context of clinical trials or well‐designed prospective registries.^9^ LIBERATE is a prospective clinical registry of patients who have undergone focal LDR brachytherapy for low‐intermediate‐risk prostate cancer. This study aimed to assess functional outcomes and treatment‐related toxicity in this patient cohort.

## 2 PATIENTS AND METHODS

### 2.1 Study design

LIBERATE is an ongoing, prospective, single‐arm, multi‐centre IDEAL Stage 2b investigator‐led clinical registry of men treated with focal LDR brachytherapy for low‐ to intermediate‐risk prostate cancer since September 2019 (ACTRN:12619001669189). The study was approved by the Bellberry Human Research Ethics Committee (Ref 2019–09‐807). Men were eligible for focal LDR brachytherapy in the LIBERATE registry if they were 40–85 years old, had a life expectancy >10 years, PSA < 15 ng/ml, clinical stage T1c/T2a, PIRADS 3–5 or a suspicious lesion on PSMA‐PET, imaging‐concordant ISUP GG1 (≥10 mm in ≥1 core), ISUP GG2 (any length) or ISUP GG3 (longest core <10 mm), with template biopsies showing no cancer or clinically insignificant cancer (ISUP GG1 < 10 mm). This analysis included all registry patients with at least 12 months of follow‐up who completed three or more patient‐reported outcome measures (PROMs) questionnaires evaluating functional outcomes across different time points.

### 2.2 Technique

The methodology has been extensively described in our prior work.^10^ All patients were treated with focal LDR brachytherapy by a single senior radiation oncologist using a three‐phase implant approach: pre‐planning volumetric assessment, seed implantation and post‐implantation dosimetry. Pre‐planning transrectal ultrasound (TRUS) and multi‐parametric magnetic resonance imaging (mpMRI) were fused using VariSeed (Varian Medical Systems, CA) and proofread by an experienced radiation therapist or medical physicist. The focal gross tumour volume (F‐GTV) represented the radiological extent of the index lesion, while the focal planning target volume (F‐PTV) included a 7 mm isotropic safety margin.

The brachytherapy seeds were inserted under general anaesthesia in the extended lithotomy position with real‐time dosimetric analysis using the VariSeed suite. Iodine‐125 seeds (Amersham model 6711‐equivalent) were placed under TRUS guidance, with additional ‘voodoo’ seeds used to ensure a 145 Gy dose to the F‐PTV. A flexible cystoscopy was performed at the end of the procedure to check for any seeds in the urethra or bladder. A rectal spacer was injected for posterior lesions. Post‐implant dosimetry included non‐contrast pelvic Computed Tomography (CT) co‐registered with same‐day mpMRI 4 weeks after implantation.

### 2.3 Follow up

Follow‐up was conducted 6 weeks post‐treatment, every 3 months for the first 2 years, and then every 6 months up to 5 years after treatment. The review included a clinical examination, serial PSA measurement and evaluation of any treatment‐related toxicities. Adverse events were clinically graded according to the National Cancer Institute (NCI) Common Terminology Criteria for Adverse Events (CTCAE), version 5.0. The protocol involved routine surveillance mpMRI and repeat transperineal prostate biopsy 18–24 months after seed implantation. Functional outcomes were assessed using validated instruments, including the International Prostate Symptom Score (IPSS), the Expanded Prostate Cancer Index Composite (EPIC) bowel score, and the five‐item version of the International Index of Erectile Function (IIEF‐5). Patients completed PROM questionnaires at baseline, 6 weeks, and then six‐monthly thereafter. Based on prior studies, the validated minimal important differences (MIDs) were ±3.1 points for IPSS,^11^ ±5 points for EPIC bowel score,^12^ and ±4 points for IIEF‐5 score.^13^ Potency was defined as an IIEF‐5 score >11.^14^, ^15^ Data was prospectively gathered and quality‐controlled by research coordinators at the Icon Cancer Centre.

### 2.4 Statistical analysis

The visual inspection of data distributions was used to evaluate normality. Continuous variables were reported as means with standard deviation (SD) or medians with interquartile range (IQR), depending on distribution, while categorical variables were expressed as counts and percentages. The Wilcoxon signed‐rank test was employed to compare functional PROM scores at each follow‐up episode with baseline. Score differences from baseline were also evaluated against the validated MIDs. Violin plots illustrated the distributions of IPSS, EPIC bowel and IIEF‐5 scores. Analyses of erectile outcomes were stratified by IIEF‐5 > 16 (no/mild ED) versus ≤16 (mild to severe ED) to reflect clinical counselling needs and to better characterise outcomes according to baseline function. Comparisons between patients with and without GU or GI adverse events (AEs) were performed using Student's t‐test or Mann–Whitney U test for continuous variables and Pearson's Chi‐square or Fisher's exact test for categorical variables, as appropriate. Significant factors from univariate analysis were included in the multivariate logistic regression to predict GU AEs. GU or GI AEs were visualised on a stacked bar chart stratified by follow‐up encounters. The Kaplan–Meier failure function was used to evaluate and depict the cumulative incidence of Grade ≥2 GU or GI AEs. For patients with multiple AE episodes, the time to the first episode was recorded as the time to failure. A statistician from Monash University independently reviewed all analyses and interpretations. Statistical tests were performed using Stata 18.0 (StataCorp, TX), with two‐sided *p* < 0.05 deemed statistically significant.

## 3 RESULTS

### 3.1 Baseline characteristics

Of 120 men enrolled in the registry, 88/120 (73.3%) had a follow‐up of ≥12 months, of whom 79/88 (89.7%) completed PROM questionnaires. The remaining 32 patients (26.7%) all had <12 months of follow‐up and were therefore not included in this analysis. The median (IQR) patient follow‐up was 33 (26–41) months (Table 1). The cohort had a mean ± SD age of 70.9 ± 6.7 years. Based on IIEF‐5 scores at baseline, 40 (50.6%) men had no or mild erectile dysfunction (ED), 26 (32.9%) had mild to severe ED, and 13 (16.5%) were not sexually active. On baseline transperineal biopsy, 1 (1.3%) man had ISUP GG1, 66 (83.5%) had ISUP GG2, and 12 (15.2%) had ISUP GG3 disease. None of the patients received androgen deprivation therapy (ADT) or 5α‐Reductase inhibitors for gland size reduction as part of focal LDR brachytherapy treatment.

**TABLE 1:** Baseline characteristics.

Variable	Value
Number of patients, *n*	79
Follow up (months), median (IQR)	33 (26–41)
Age at brachytherapy (years), mean ± SD	70.9 ± 6.7
BMI (kg/m^2^), mean ± SD	27.4 ± 3.6
Prostate volume (cc), median (IQR)	37 (29–50)
Previous TURP, *n* (%)	5 (6.3)
Baseline ED based on IIEF‐5 score, *n* (%)	
No to mild ED (17–25)	40 (50.6)
Mild to severe ED (5–16)	26 (32.9)
Not sexually active	13 (16.5)
Comorbidity, *n* (%)	
Hypertension	32 (40.5)
Diabetes	9 (11.4)
Smoking status, *n* (%)	
Current smoker	4 (5.1)
Ex‐smoker	20 (25.3)
Never smoked	55 (69.6)
Baseline PSA (ng/mL), mean ± SD	6.0 ± 2.6
Clinical stage, *n* (%)	
T1c	69 (87.3)
T2a	10 (12.7)
PIRADS score, *n* (%)	
2 and suspicious lesion on PSMA PET	2 (2.5)
3	5 (6.3)
4	58 (73.4)
5	13 (16.5)
No MRI but suspicious PSMA PET	1 (1.3)
Lesion location, *n* (%)^a^	
Anterior	40 (28.6)
Mid	43 (30.7)
Posterior	57 (40.7)
Baseline transperineal biopsy, *n* (%)	
ISUP GG1	1 (1.3)
ISUP GG2	66 (83.5)
ISUP GG3	12 (15.2)

Abbreviations: BMI, body mass index; ED, erectile dysfunction; IQR, interquartile range; ISUP GG, International Society of Urological Pathology grade group; MRI, magnetic resonance imaging; *n*, number; PIRADS, prostate imaging‐reporting and data system; PSA, prostate‐specific antigen; PSMA PET, prostate‐specific membrane antigen positron emission tomography; SD, standard deviation; TURP, transurethral resection of the prostate.

^a^ Several lesions crossed into multiple zones.

### 3.2 Dosimetry outcomes

Post‐implantation dosimetric outcomes are listed in Table 2. The median (IQR) number of seeds used was 28 (23–33), with a mean ± SD total implanted activity of 10.8 ± 3.3 mCi. The median F‐PTV was 7.2 cc (range 2.6–20.2 cc), and the median F‐PTV to total prostate volume ratio was 18.2% (range 6.0%–81.0%). For the anterior rectal wall, the median volume exposed to V100% was 0.00 cc (range 0.00–0.50 cc), with a median maximum dose of 47.3 Gy (range 15.7–142.5 Gy).

**TABLE 2:** Dosimetry outcomes.

Variable	Value
Rectal spacer insertion, *n* (%)	72 (91.1)
Number of seeds, median (IQR)	28 (23–33)
Total implanted activity (mCi), mean ± SD	10.8 ± 3.3
Geometry, median [range]	
Prostate volume (cc)	37 [11–107]
F‐GTV (cc)	1.7 [0.2–8.3]
F‐PTV (cc)	7.2 [2.6–20.2]
F‐PTV (% of prostate volume)	18.2 [6.0–81.0]
F‐GTV, median [range]	
V100% (%)	100 [92.7–100]
V150% (%)	97.7 [52.0–100]
D90% (Gy)	259.8 [150.5–447.5]
Prostate, median [range]	
V100% (%)	22.1 [6.7–71.5]
Urethra, median [range]	
Max (Gy)	170.9 [39.9–468.9]
V200% (cc)	0.00 [0.00–0.14]
Rectum, median [range]	
Max (Gy)	47.3 [15.7–142.5]
V100% (cc)	0.00 [0.00–0.50]

Abbreviations: D90%, dose to 90% of the structure volume; F‐GTV, focal gross tumour volume; F‐PTV, focal planning target volume; Gy, Gray; IQR, interquartile range; mCi, millicurie; n, number; SD, standard deviation; V100%, volume receiving 100% of the prescribed dose; V150%, volume receiving 150% of the prescribed dose; V200%, volume receiving 200% of the prescribed dose.

### 3.3 IPSS

**FIGURE 1:**
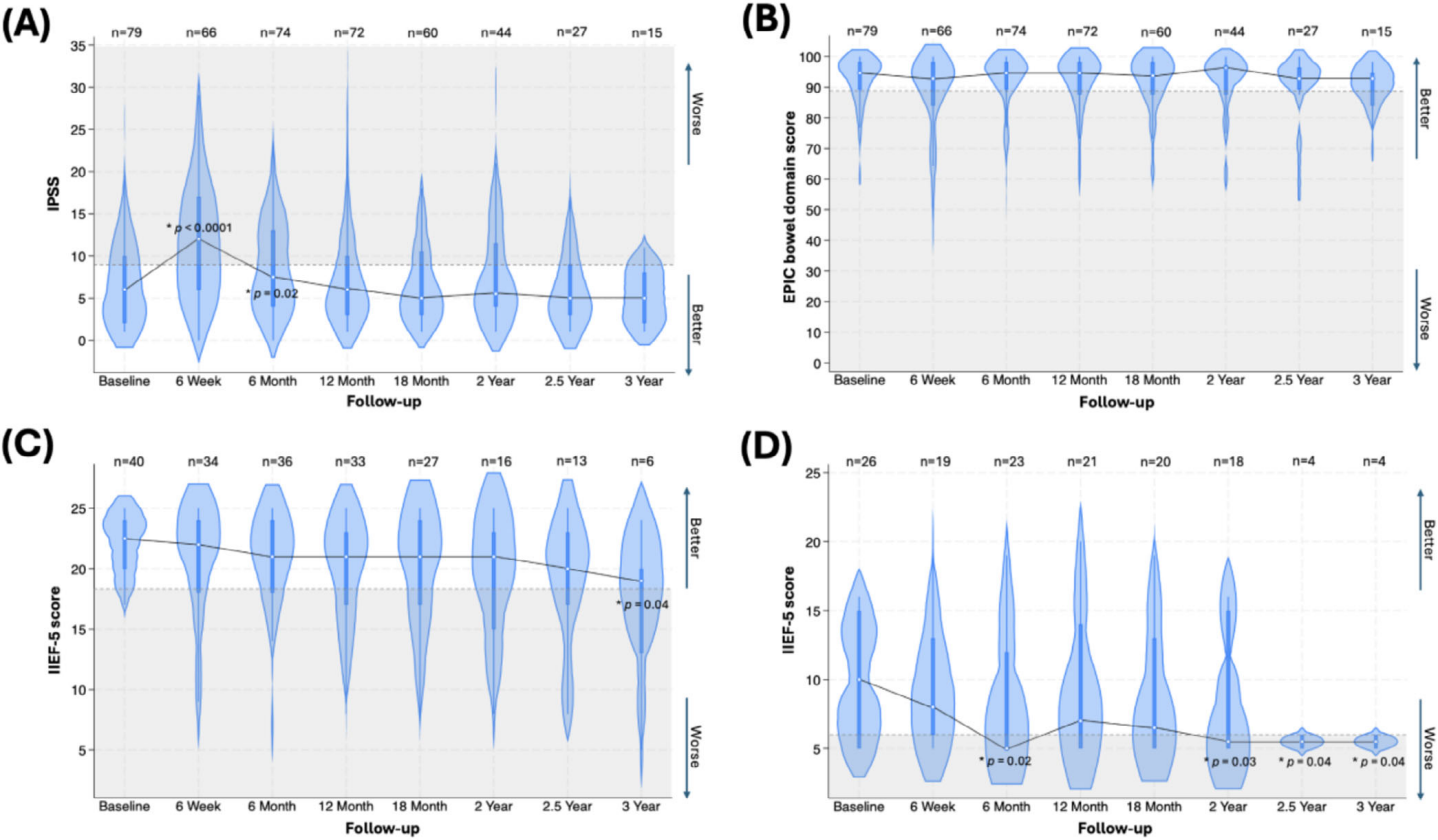
Violin plots with embedded box plots depicting patient‐reported outcome measures (PROMs) for genitourinary, gastrointestinal, and erectile functions at various time points relative to focal LDR brachytherapy. (A) International prostate symptom score (IPSS), (B) the expanded prostate cancer index composite (EPIC) bowel score, (C) international index of erectile function (IIEF‐5) score in patients with no or mild ED at baseline (IIEF‐5 17–25), (D) IIEF‐5 score in patients with mild to severe ED at baseline (IIEF‐5 5–16). The dotted grey line indicates the threshold for minimal important difference (MID), with the grey‐shaded area representing the range of clinically meaningful changes from baseline. Validated MID values: 3.1 for IPSS, ±5 for EPIC bowel domain, and ±4 for IIEF‐5. The central white dot shows the median, the thick bar in the Centre indicates the interquartile range (IQR), and the whiskers extend to 1.5 times the IQR. Violin plots may extend beyond minimum and maximum values due to the smoothing effect of kernel density estimation. The width of the blue plot at different values reflects the data density, offering a detailed visualisation of the distribution. *Statistically significant change from baseline.

The median (IQR) IPSS was 6 (2–10) at baseline, rising to 12 (6–17) at 6 weeks and then recovering to 7.5 (4–13) at 6 months, eventually returning to the baseline value of 6 (3–10) at 12 months. While the increases in IPSS at 6 weeks and 6 months compared to baseline were statistically significant, only the initial increase at 6 weeks (+6 points) met the MID criteria for clinical significance. The temporal pattern was not different when stratified by the baseline IPSS category. From 18 months onward, IPSS demonstrated a minor improvement compared to baseline (Figure 1A), although this change was neither statistically significant nor clinically meaningful.

### 3.4 EPIC bowel score

Figure 1B illustrates that the EPIC bowel score remained relatively stable throughout the follow‐up period, with scores clustering near the upper end of the scale. Given spacer use in 91.1%, our bowel functional outcomes reflect the combined effect of focal LDR therapy and rectal spacer use. Exploratory spacer versus no‐spacer analyses were underpowered and hypothesis‐generating only. The median (IQR) EPIC bowel score was 94.6 (89.3–98.2) at baseline, 92.9 (83.9–98.2) at 6 weeks, 94.6 (89.3–98.2) at 6 months, and 94.6 (87.5–98.2) at 12 months, with subsequent scores as detailed in Table 3. None of these changes were statistically significant or clinically meaningful.

**TABLE 3:** Functional outcomes.

		IPSS			EPIC bowel score			IIEF‐5 (baseline score 17–25)					IIEF‐5 (baseline score 5–16)			
	*n*	Med (IQR)	CFB	*n*	Med (IQR)	CFB	*n*	Med (IQR)	CFB	Pot, *n* (%)	PDEi, *n* (%)	*n*	Med (IQR)	CFB	Pot, *n* (%)	PDEi, *n* (%)
Baseline	79	6 (2–10)	0	79	94.6 (89.3–98.2)	0	40	22.5 (20–24)	0	40 (100)	4 (10.0)	26	10 (5–15)	0	10 (38.5)	0 (0)
6‐week	66	12* (6–17)	+6^a^	66	92.9 (83.9–98.2)	−1.7	34	22 (18–24)	−0.5	25 (73.5)	3 (8.8)	19	8 (6–13)	−2	5 (26.3)	0 (0)
6‐month	74	7.5* (4–13)	+1.5	74	94.6 (89.3–98.2)	0	36	21 (18–24)	−1.5	29 (80.6)	8 (27.6)	23	5* (5–12)	−5^a^	5 (21.7)	3 (13.0)
12‐month	72	6 (3–10)	0	72	94.6 (87.5–98.2)	0	33	21 (17–23)	−1.5	28 (84.8)	3 (10.7)	21	7 (5–14)	−3	5 (23.8)	7 (33.3)
18‐month	60	5 (3–10.5)	−1	60	93.8 (87.5–98.2)	−0.8	27	21 (17–24)	−1.5	23 (85.2)	5 (21.7)	20	6.5 (5–13)	−3.5	5 (25.0)	8 (40.0)
2‐year	44	5.5 (4–11.5)	−0.5	44	96.4 (87.5–96.5)	+1.8	16	21 (15–23)	−1.5	13 (81.3)	5 (38.5)	18	5.5* (5–15)	−4.5^a^	4 (22.2)	4 (22.2)
2.5‐year	27	5 (3–9)	−1	27	92.9 (89.3–96.4)	−1.7	13	20 (17–23)	−2.5	11 (84.6)	5 (45.5)	4	5.5* (5–6)	−4.5^a^	0 (0)	0 (0)
3‐year	15	5 (2–8)	−1	15	92.9 (94.6–83.9)	−1.7	6	19* (13–20)	−3.5	5 (83.3)	2 (40.0)	4	5.5* (5–6)	−4.5^a^	0 (0)	1 (25.0)

*Note*: The time points are in reference to the focal brachytherapy procedure.

Abbreviations: CFB, change from baseline; EPIC, Expanded Prostate Cancer Index Composite; IIEF‐5, five‐item version of the International Index of Erectile Function; IPSS, International Prostate Symptom Score; IQR, interquartile range; Med, median; n, number; PDEi, Phosphodiesterase Inhibitors; Pot, potency (IIEF‐5 > 11).

^a^ Clinically significant change from baseline, met the minimal important difference (MID) criteria in the respective domain. The validated MID was ±3.1 points for IPSS, ±5 points for EPIC bowel score, and ±4 points for IIEF‐5 score.

* *p* < 0.05, a statistically significant change in PROM score compared with the baseline level in the respective domain.

### 3.5 IIEF‐5 score: Entire cohort of sexually active men

Across all 66 sexually active men, focal LDR brachytherapy was not associated with a statistically significant or clinically meaningful change in median IIEF‐5 scores compared with baseline. To further explore the impact of treatment in men with differing baseline function, we stratified the analysis by IIEF‐5 > 16 (no/mild ED) versus ≤16 (mild to severe ED).

### 3.6 IIEF‐5 score: No or mild ED (IIEF‐5 17–25) at baseline

Of these 66 men, 40 (60.6%) had no or mild ED at baseline (IIEF‐5 score 17–25). Their median (IQR) IIEF‐5 score was 22.5 (20–24) at baseline, decreasing to 22 (18–24) at 6 weeks, and plateauing at 21 (18–24) at 6 months (Figure 1C). The median (IQR) IIEF‐5 score further declined to 20 (17–23) at 2 years, 20 (17–23) at 2.5 years, and 19 (13–20) at 3 years, although these follow‐up assessments included fewer patients (n < 20). While the decline in IIEF‐5 score at 3 years was statistically significant, none of the changes met the criteria for clinical significance. Potency rates initially declined at 6 weeks post‐treatment but largely recovered by 12 months (Table 3).

### 3.7 IIEF‐5 score: Mild to severe ED (IIEF‐5 5–16) at baseline

Among the 26 patients with mild to severe ED at baseline (IIEF‐5 score 5–16), the median (IQR) IIEF‐5 score was 10 (5–15) at baseline, decreasing to 8 (6–13) at 6 weeks and 5 (5–12) at 6 months. The score then partially recovered to 7 (5–14) at 12 months before declining again to 6.5 (5–13) at 18 months, 5.5 (5–15) at 2 years, and 5.5 (5–6) at 2.5 and 3 years. The declines observed at 6 months and from 2 years onward were both statistically significant and clinically meaningful. Figure 1D further illustrates a higher frequency of scores clustering at the lower end of the IIEF‐5 scale. Potency rates declined considerably at 6 weeks post‐treatment but did not recover to near‐baseline levels at subsequent time points.

### 3.8 Toxicity as per CTCAE v5.0

**FIGURE 2:**
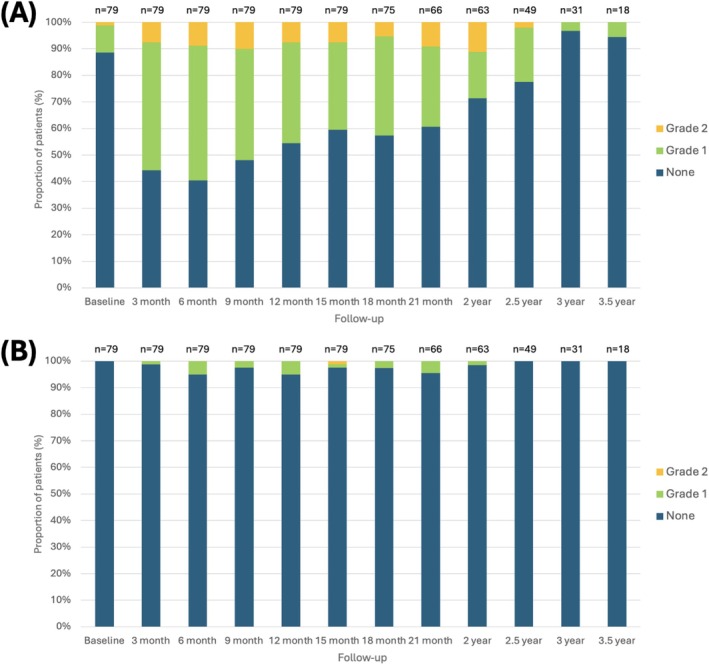
Cumulative incidence curves with 95% confidence intervals for grade ≥2 genitourinary (GU) and gastrointestinal (GI) adverse events. For patients with multiple grade ≥2 toxicities, the time to failure was defined as the time to the first grade ≥2 adverse event. The time axis is measured relative to the focal brachytherapy procedure.

**FIGURE 3:**
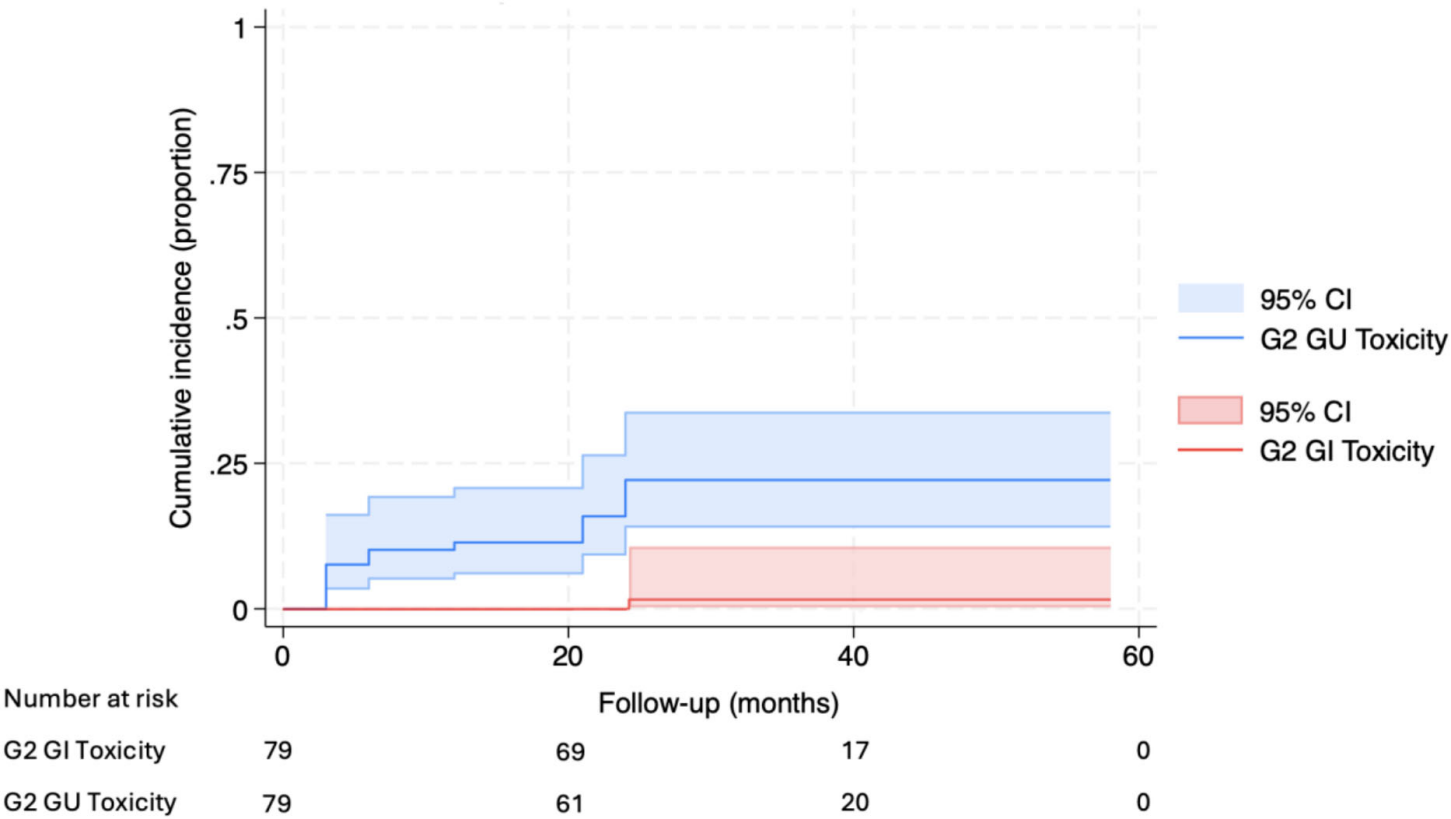
Stacked bar charts illustrating toxicity grades per CTCAE v5.0 at various time points relative to the focal LDR brachytherapy procedure. (A) Genitourinary adverse events. (B) Gastrointestinal adverse events. CTCAE: common terminology criteria for adverse events. A single patient may have reported the same adverse event at multiple time points.

A total of 16 patients (20.3%) reported Grade 2 GU AEs, while one (1.3%) experienced Grade 2 GI AEs. No Grade ≥3 GI or GU AEs were observed. The cumulative incidences of Grade 2 GU or GI AEs are illustrated in Figure 2. The frequency and severity of GU AEs peaked at 3–12 months, with subsequent improvement (Figure 3A). Temporary Grade 1 obstructive and irritative lower urinary tract symptoms (LUTS) were the most common AEs (Table 4). GI AEs were infrequent, and nearly all were Grade 1 (Figure 3B).

**TABLE 4:** Adverse events according to CTCAE v5.0.

	Acute (≤3 months)		Late (>3 months)	
	Grade 1	Grade 2	Grade 1	Grade 2
**Genitourinary toxicities**	*n* (%)	*n* (%)	*n* (%)	*n* (%)
Cystitis	1 (1.3)	–	3 (3.8)	–
Haematuria	1 (1.3)	–	3 (3.8)	3 (3.8)
Urinary fistula	–	–	–	–
Urinary frequency	12 (15.2)	4 (5.1)	35 (44.3)	10 (12.7)
Urinary urgency	15 (19.0)	2 (2.5)	26 (32.9)	7 (8.9)
Urinary incontinence	–	–	5 (6.3)	3 (3.8)
Urinary retention	2 (2.5)	–	13 (16.5)	2 (2.5)
Obstructive LUTS	7 (8.9)	1 (1.3)	27 (34.2)	5 (6.3)
Genitourinary pain	6 (7.6)	–	13 (16.5)	3 (3.8)
Perianal or scrotal pain	1 (1.3)	–	3 (3.8)	1 (1.3)
**Gastrointestinal toxicities**	**n (%)**	**n (%)**	**n (%)**	**n (%)**
Diarrhoea	1 (1.3)	–	7 (8.9)	1 (1.3)
Proctitis	–	–	3 (3.8)	–
**Other toxicities**	**n (%)**	**n (%)**	**n (%)**	**n (%)**
Arthralgia	–	–	1 (1.3)	–
Fatigue	2 (2.5)	–	4 (5.1)	–

*Note*: The toxicity rates reported reflect the highest‐grade adverse event in each domain, with individuals potentially experiencing multiple categories of adverse events.

Abbreviation: CTCAE, Common Terminology Criteria for Adverse Events.

### 3.9 Predictors of toxicity

In the univariate analysis, age and baseline IPSS were significant predictors of Grade 2 GU AEs. Patients with Grade 2 GU AEs were older and had a higher baseline IPSS (mean ± SD age: 73 ± 7.2 versus 70 ± 6.2, *p* = 0.04; median (IQR) IPSS: 10 (8–12) versus 5 (2–9.5), *p* = 0.003). In the multivariate analysis, baseline IPSS remained a significant predictor of Grade 2 GU AEs (OR 1.14, 95% CI 1.02–1.28, *p* = 0.02). The maximum rectal dose was the only significant predictor of Grade ≥1 GI AEs. Men who experienced Grade ≥1 GI AEs had higher maximum rectal doses than those who did not (median (IQR): 59.6 (46.5–86.1) versus 43.7 (31.1–61.7) Gy, *p* = 0.04).

## 4 DISCUSSION

With a median follow‐up of 33 months, this study demonstrated that focal LDR brachytherapy was associated with favorable functional outcomes. A transient but clinically meaningful increase in IPSS was observed, which returned to baseline by 12 months. The EPIC bowel score remained stable throughout the follow‐up. Among patients with no or mild ED at baseline, the IIEF‐5 score gradually declined over time, though these changes were not clinically significant. In contrast, men with mild to severe baseline ED experienced statistically significant and clinically meaningful declines in IIEF‐5 scores at 6 months and from 2 years onwards. Most AEs were GU, primarily transient Grade 1 LUTS, particularly in older patients with elevated baseline IPSS.

Our results align with prior research on functional outcomes following focal LDR brachytherapy.^5^, ^16^, ^17^, ^18^ Ta et al. analysed 39 men who underwent focal LDR brachytherapy and observed a significant increase in IPSS at 2 months, likely due to acute inflammatory response and prostatic swelling from seed implantation, with no statistically significant differences from baseline at later time points (mean IPSS: 6.7 at baseline, 12.5 at 2 months, and 6 at 12 months).^16^ They also observed a marked decline in IIEF‐5 scores at 2 months, with partial recovery over time, though scores did not return to baseline (mean IIEF‐5: 18 at baseline, 14.3 at 2 months, and 16.2 at 12 months). Similarly, Cosset et al., in a study of 21 patients treated with focal LDR brachytherapy, noted a sharp increase in IPSS at 2 months followed by a return to near‐baseline levels by 12 months (mean IPSS: 5.4 at baseline, 11.8 at 2 months, and 6.1 at 12 months).^17^ They also observed a minor decline in IIEF‐5 score at 2 months, which improved on subsequent visits (mean IIEF‐5: 20.1 at baseline, 18.6 at 2 months, and 19.8 at 12 months). Notably, our study achieved superior dosimetric outcomes, delivering a higher dose to F‐PTV while maintaining similar or lower doses to organs at risk (OARs) compared to previous studies.^17^, ^19^

A comparison of the few studies on focal LDR and focal HDR brachytherapy suggests that both techniques result in a similar decline in erectile function.^5^, ^16^, ^17^, ^18^, ^20^, ^21^ However, focal HDR brachytherapy has a less acute impact on IPSS, likely due to its radiobiologic advantages. Our findings support these reports. HDR brachytherapy resembles an extreme hypofractionation approach, whereas LDR brachytherapy corresponds to a more conventional fractionation regimen. Bladder tissue, with a high α/β ratio, is less sensitive to fraction size but more affected by cumulative dose, whereas prostate cancer, with a low α/β ratio, is more sensitive to fraction size than total dose.^22^ Hence, a hypofractionated approach with larger doses per fraction and a reduced total dose may reduce urinary toxicity. This advantage, however, is offset by a higher risk of urethral strictures with HDR brachytherapy, likely due to dosimetric factors and increased radiation dose to the urethra.^23^ It is also a more complex and resource‐intensive procedure and may necessitate hospital admission for a few days.

This study demonstrated that in patients with no or mild ED, potency rates initially decreased at 6 weeks post‐treatment but largely recovered by 12 months, which is consistent with the literature.^5^, ^17^, ^24^ Although focal brachytherapy negatively impacts erectile function regardless of baseline status, the effect is particularly pronounced and clinically significant in patients with mild to severe baseline ED. Similar results have also been observed with other focal therapy modalities.^7^ Wysock et al., in their study of 106 men undergoing partial gland cryoablation, highlighted that patients with moderate ED are especially vulnerable to experiencing a decline in sexual function following focal therapy.^25^ Therefore, it is crucial to set realistic expectations and communicate the increased risk of a substantial drop in erectile function to patients with mild to severe ED who are considering focal brachytherapy.

The limited literature on focal LDR brachytherapy indicates minimal serious GI toxicity, particularly when rectal spacers are used.^5^, ^16^, ^17^ Our findings support this observation, as the EPIC bowel score remained stable throughout the follow‐up. Only one patient (1.3%) experienced Grade 2 GI AEs, and no Grade ≥3 GI AEs were reported. Notably, most studies examining functional outcomes after focal brachytherapy have relied solely on clinician‐graded GI AEs, without incorporating validated PROMs to evaluate GI symptoms.^5^, ^16^, ^17^ Langley et al. used the GI component of the EORTC QLQ‐PR25 in their study of hemi‐gland LDR brachytherapy involving 30 patients.^24^ They reported peak bowel symptom scores at 6 months, with a slow return to baseline. It is worth noting that rectal spacers were not used in their study, potentially accounting for the observed poorer bowel quality‐of‐life outcomes.

Our GU and GI toxicity rates were considerably lower than those reported for whole‐gland LDR brachytherapy, which is associated with approximately 5%–8% Grade ≥3 GU AEs and 0.5%–1% Grade ≥3 GI AEs.^26^, ^27^ No Grade ≥3 GI or GU AEs were observed in our cohort. This reduction in toxicity is likely attributable to the smaller proportion of the prostate irradiated, which inherently lowers the risk of toxicity. The median F‐PTV in our study accounted for 18.2% of the prostate volume (range 6.0%–81.0%). It is reasonable to expect that true focal brachytherapy would result in lower toxicity rates and improved functional outcomes compared to hemi‐gland brachytherapy. Although our findings are broadly similar to the two existing studies on hemi‐gland LDR brachytherapy, significant heterogeneity in patient selection, treatment protocols and baseline urinary and erectile function among these studies makes direct comparisons challenging.^24^, ^28^ Nevertheless, the observed toxicity rates across these studies, including ours, demonstrate a consistent trend, reinforcing the potential of focal and hemi‐gland brachytherapy to reduce treatment‐related morbidity compared with whole‐gland brachytherapy in highly selected patients.

To our knowledge, this is the largest study to date on functional outcomes following focal brachytherapy. Key strengths include its prospective design and comprehensive assessment of functional outcomes using both clinician‐graded and patient‐reported outcome measures. Nonetheless, this study has several limitations, such as its non‐randomised, single‐arm design and relatively small sample size. High spacer utilisation limits the strength of subgroup analyses, as spacer‐free numbers were too small for reliable comparison. Furthermore, although the follow‐up period is longer than that of many comparable studies, it may still be inadequate for capturing certain late toxicities.

In conclusion, with a median follow‐up of 33 months, these findings demonstrate that focal LDR brachytherapy is associated with favourable functional outcomes in patients with low‐intermediate risk prostate cancer. Future integration of focal LDR brachytherapy into clinical practice will require: (i) reproducible technical protocols with standardised safety margins and dosimetric constraints to reliably target index lesions while minimising dose to organs at risk; (ii) refined selection criteria to identify patients in whom definitive treatment is both necessary (based on clinical, pathological or molecular risk factors) and achievable with focal therapy; and (iii) robust evaluation of long‐term oncological control against both active surveillance and radical treatment in prospective comparative studies. These steps, framed against the trajectory of other focal modalities, will determine whether focal LDR brachytherapy evolves from promising tolerability to an established component of prostate cancer management.

## AUTHOR CONTRIBUTIONS

Conceptualisation and study design: Mohammadmehdi Adhami, Jeremy Cheng, Elliot Anderson, Lloyd Smyth, Nathan Lawrentschuk, Jeremy Grummet and Andrew See. Data acquisition: Mohammadmehdi Adhami, Elliot Anderson, Lloyd Smyth, Cate Davey, Thang Nguyen, Jeremy Grummet and Andrew See. Data analysis and interpretation: Mohammadmehdi Adhami and Jeremy Grummet. Manuscript writing: Mohammadmehdi Adhami. Manuscript review: Mohammadmehdi Adhami, Jeremy Cheng, Elliot Anderson, Lloyd Smyth, Cate Davey, Thang Nguyen, Richard O'Sullivan, Andrew Ryan, Nathan Lawrentschuk, Jeremy Grummet and Andrew See. All authors have reviewed the manuscript and approved the final version for publication.

## CONFLICT OF INTEREST STATEMENT

The authors declare no conflicts of interest.
